# Serotype Distribution and Antimicrobial Susceptibility Pattern of *Streptococcus pneumoniae* in COVID-19 Pandemic Era in Brazil

**DOI:** 10.3390/microorganisms12020401

**Published:** 2024-02-17

**Authors:** Samanta C. G. Almeida, Ana Paula S. de Lemos, Ana Luiza Bierrenbach, José Cássio de Moraes, Maria Cristina de Cunto Brandileone

**Affiliations:** 1 Center of Bacteriology, National Laboratory for Meningitis and Invasive Pneumococcal Infections, Institute Adolfo Lutz, São Paulo 01246-902, Brazil; ana.lemos@ial.sp.gov.br (A.P.S.d.L.); maria.brandileone@ial.sp.gov.br (M.C.d.C.B.); 2 Hospital Sírio-Libanês, Institute of Education and Researcher, São Paulo 01308-060, Brazil; albierrenbach@yahoo.com.br; 3 Santa Casa of São Paulo School of Medical Sciences, São Paulo 01224-001, Brazil; jcassiom63@gmail.com

**Keywords:** *Streptococcus pneumoniae*, invasive pneumococcal disease, pneumococcal serotypes, pneumococcal vaccines

## Abstract

Despite the introduction of the pneumococcal vaccine, *Streptococcus pneumoniae* remains a cause of invasive diseases in Brazil. This study provides the distribution of serotypes and antimicrobial susceptibility patterns for pneumococcal isolates before and during the years of the COVID-19 pandemic in two age groups, <5 and ≥50 years. This is a national laboratory-based surveillance study that uses data from the Brazilian national laboratory for invasive *S. pneumoniae* from the pre-COVID-19 (January 2016 to January 2020) and COVID-19 (February 2020 to May 2022) periods. Antimicrobial resistance was evaluated by disk diffusion and minimum inhibitory concentration. The year 2020 was marked by a 44.6% reduction in isolates received and was followed by an upward trend from 2021 onwards, which became evident in 2022. No differences were observed in serotypes distribution between the studied periods. The COVID-19 period was marked by the high prevalence of serotypes 19A, 3, and 6C in both age groups. Serotypes 19A and 6C were related to non-antimicrobial susceptibility. We observed a reduction in *S. pneumoniae*, without changes in serotypes distribution and epidemiological capsular switch during the COVID-19 period. We observed elevated resistance rates, mainly to penicillin and ceftriaxone for non-meningitis cases in children under 5 years of age.

## 1. Introduction

Despite the widespread use of pneumococcal conjugate vaccines (PCVs) in immunization programs, *S. pneumoniae* continues to cause severe invasive diseases such as meningitis, sepsis, and bacteremic pneumonia, which is associated with high morbidity and mortality worldwide among all age groups, especially among young children and the elderly [1].

*S. pneumoniae* is a respiratory-borne pathogen that colonizes the upper respiratory tract of humans. Its transmission occurs through close contact between people and through respiratory tract material, such as saliva droplets or aerosols. *S. pneumoniae* also has several virulence factors that are crucial for its transmission, colonization, and invasion [1]. One of these factors is the polysaccharide capsule, which defines pneumococcal serotypes, induces serotype-specific protective immunity, and is, therefore, the antigen of the pneumococcal vaccines. To date, around 100 pneumococcus serotypes have been described, all of which have the potential to cause disease, although some are more invasive than others [2]. Currently available pneumococcal vaccine formulations contain multiple prevalent antigens from 10, 13, 15, 20, and 23 serotypes [3,4,5,6,7]. In Brazil, the 10-valent PCV was introduced in the national childhood immunization program in 2010 [8] and is still used in routine immunization [9].

The efforts to control the COVID-19 pandemic interfered with the dynamics of transmission of respiratory diseases, modifying the seasonality of *S. pneumoniae* respiratory diseases associated with an elevated incidence in the winter periods. Non-pharmacological measures to control COVID-19 have deeply reduced *S. pneumoniae* transmission and contagion, changing the global epidemiology of invasive pneumococcal disease (IPD) [10]. In Brazil, from the pre-COVID period (2016 to 2019) to the pandemic period (considering the years 2020 and 2021), the average annual incidence of confirmed pneumococcal meningitis declined from 0.47 to 0.17 per 100,000 inhabitants, according to data from the Notifiable Diseases Information System. In 2022, the levels returned to the pre-COVID levels, reaching 0.54 per 100,000 inhabitants (Supplementary Figure S1).

Bacterial co-infection in patients with SARS-CoV-2 appears to be uncommon, such as that caused by *S. pneumoniae* [11,12]. The empirical use of antibiotics as a therapeutic strategy, particularly azithromycin, largely used in hospitalized patients with COVID-19 in Brazil [12,13], could have had an impact on increasing resistance to antibiotics.

Given the progress of the COVID-19 pandemic in Brazil and its impact on IPD, the present study aimed to assess the distribution of serotypes and antimicrobial susceptibility patterns for pneumococcal isolates before and during the years of the COVID-19 pandemic, exploring data from the Brazilian national laboratory surveillance for *Streptococcus pneumoniae*.

## 2. Materials and Methods

Supplementary Figure S2 summarizes the methodology used in this study.

### 2.1. Study Design and Population

This is a national laboratory-based surveillance study conducted from January 2016 to May 2022 using data from the national laboratory for public health surveillance of IPD in Brazil. *S. pneumoniae* isolates recovered from normally sterile clinical specimens (mainly for cerebrospinal fluid, blood, and pleural fluid) were collected in public health laboratories and hospitals across the country and were routinely sent in a culture medium (blood agar or chocolate agar) to the Centre of Bacteriology at the Institute Adolfo Lutz (IAL), the Brazilian National Reference Laboratory for meningitis and IPD. This network is coordinated by the Brazilian Ministry of Health and encompasses 26 public health laboratories located in each of the Brazilian states, covering the whole country [14]. The isolates were sent to the IAL, along with data on age, gender, clinical diagnosis of the patient, and municipality where the sample was collected.

### 2.2. Microbiology Methods

All pneumococcal IPD isolates were confirmed at the IAL using standard methods [15]. Briefly, the *S. pneumoniae* isolates were cultured on sheep blood agar plates for 18–24 h on 5% CO_2_. The species were identified by the susceptibility on the optochin test and the solubility on the bile solubility test. *S. pneumoniae* were serotyped by means of the Pneumotest-latex agglutination kit and the Quellung reaction using antisera, both from the Statens Serum Institute (Copenhagen, Denmark). Non-typeable (NT) isolates identified by Quellung were also verified by sequential multiplex PCR; deduction of serotypes/serogroups was achieved using 41 primer pairs for serotype/serogroup discrimination plus a primer pair for *S. pneumoniae* capsule identification (*cps* gene) using nine sequential reactions following the gene targets and protocols described by the Center for Diseases Control and Prevention (CDC), Atlanta, GA, USA [16,17]. In summary, the reactions were performed on eight sequential reactions (each with five primer pairs to serotype/serogroup plus the *cps* gene primer) and one reaction to discriminate serogroup 6 (with two primer pairs to identify serotypes 6A/B and 6C/D plus the *cps* gene primer).

Susceptibility to oxacillin 1 μg (screening for susceptibility to penicillin and ceftriaxone), erythromycin 15 μg, clindamycin 2 μg, vancomycin 30 μg, levofloxacin 5 μg, sulfamethoxazole-trimethoprim 23.75–1.25 μg, and tetracycline 30 μg was assessed by means of the disk diffusion method as recommended by the Clinical Laboratory Standards Institute guidelines (CLSI) [18,19,20]. Isolates presenting with oxacillin resistance (zone of inhibition ≤ 19 mm) were analyzed for their minimum inhibitory concentration (MIC) by broth microdilution to penicillin (8–0.015 μg/mL) and ceftriaxone (4–0.003 μg/mL). The *S. pneumoniae* ATCC49619 strain was used as a reference. Interpretative breakpoints to antimicrobial agents (susceptible, intermediate, or resistant) were based on the CLSI guidelines of 2022 [18]. Intermediate and resistant isolates were defined as non-susceptible. Isolates resistant to at least three classes of antibiotics were defined as multidrug-resistant (MDR).

### 2.3. Data Analysis

For this investigation, data on *S. pneumoniae* isolates, associated serotypes, and antimicrobial resistance were extracted from the IAL database in Brazil. Duplicate isolates of *S. pneumoniae* from the same patient were excluded from the analysis. 

The distribution of serotypes and the susceptibility rates to antimicrobials were evaluated in two study periods, the pre-COVID-19 period (January 2016–January 2020) and the COVID-19 pandemic period (February 2020–May 2022), in two age groups, <5 and ≥50 years old. Non-susceptibility to penicillin and ceftriaxone was analyzed considering meningitis and non-meningitis.

The proportion of serotypes included in each PCV (PCV10, PCV13, PCV15, PCV20, and PCV24) was calculated by the proportion of isolates included in each formulation divided by the total number of invasive isolates, by age group and study period. 

Hypothesis testing comparing changes between the COVID-19 period and the pre-COVID-19 reference period in the proportion of serotypes included in the vaccine formulations or the proportion of non-susceptibility to antimicrobials was performed with the chi-square test or Fisher’s exact when appropriate. Given that 30 separate hypothesis tests were carried out in this study, to counteract the problem of multiple comparisons, the Holm–Bonferroni method was used. The K index identified by the method was 0.002, that is, only *p* values equal to or smaller than this value rejected the null hypothesis. It is important to highlight that, although the Holm–Bonferroni method is considered “uniformly” more powerful than the classical Bonferroni correction, it is still a conservative test; therefore, discretion is recommended in interpreting the *p*-values presented.

### 2.4. *Ethical Aspects*

The *S. pneumoniae* isolates’ data studied here came from a retrospective collection of bacterial isolates gathered by the Institute Adolfo Lutz as part of national routine surveillance activities. Patient information was obtained through routine clinical care procedures, and, during this study’s analysis, patient identification was completely anonymous. No human or animal tissue or any other biological material was used in this study. This study was approved by the Institutional Review Board, Technical and Scientific Council (CTC40N-2021) of the Institute Adolfo Lutz (São Paulo, Brazil). According to law No. 11794/2008, this study does not require evaluation from the Institute Adolfo Lutz’s Independent Ethics Committee (CEPIAL) or the Independent Ethics Committee on the Use of Experimental Animals of the Institute Adolfo Lutz (CEUA/IAL) since it did not involve any research with human beings or animals and has no ethical implications.

## 3. Results

### 3.1. S. pneumoniae Sample

From January 2016 to May 2022, the IAL received a total of 4766 *S. pneumoniae* isolates of IPD. Of those, 287 (6%) were duplicate isolates from the same patient, plus 1708 (35.8%) isolates from the age group 5 to 49 years old were excluded from the analysis. Therefore, 2771 *S. pneumoniae* isolates were included in this study, and each was classified as an IPD case; 2004 (72.3%) were classified in the pre-COVID-19 period and 767 (27.7%) in the COVID-19 period. A total of 952 (34.4%) isolates were classified in the <5-year-old and 1819 (65.6%) in the ≥50-year-old group. A total of 884 (31.9%) isolates were from meningitis cases, and 1887 (68.1%) were from non-meningitis cases (Supplementary Table S1). 

**Figure 1:**
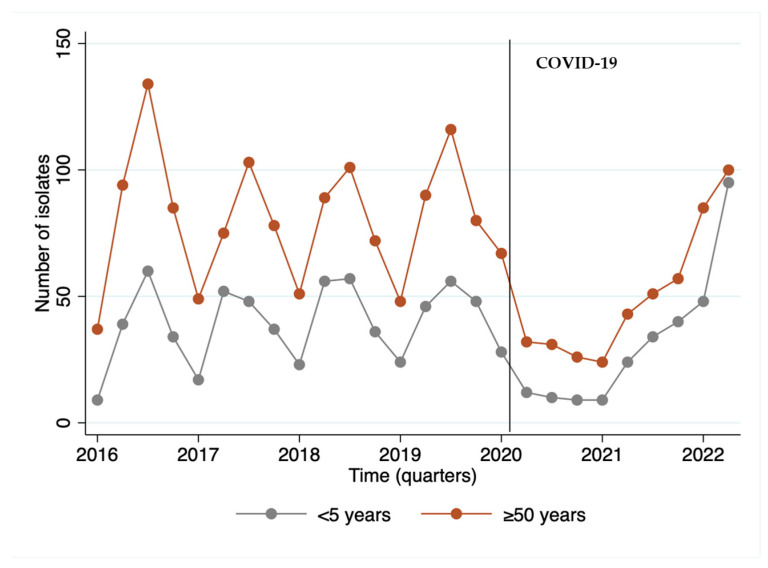
Number of invasive *S. pneumoniae* isolates (N = 2.771) per quarter of the period from 2016 to May of 2022 by age group.

We observed that the year 2020, the beginning of the COVID-19 period, was marked by a reduction of 44.6% in the invasive isolates received by the IAL, with a disruption in seasonality, followed by an upward trend starting in 2021 and becoming more evident in 2022. Remarkably, the largest number of isolates from children occurred in the last quarter of the series, when post-COVID-19 cases resumed (Figure 1).

### 3.2. Serotype Distribution 

The diversity of the serotypes is displayed by the presence of 62 serotypes associated with IPD, 60 in the pre-COVID-19 period and 50 in the COVID-19 period. The distribution according to study periods and age group is presented for the <5-year-old group (Table 1) and the ≥50-year-old group (Table 2). There was no significant change between the COVID-19 period and the reference pre-COVID-19 period in the proportion of serotypes included or not in the PCV10 vaccine. In both age groups and both periods, serotypes 19A, 3, and 6C were those with the greatest contributions. When analyzed separately, there was a statistically significant increase in the contribution of serotype 19A between the periods for children aged 5 and under (from 37.2% to 48.1%; *p*-value: 0.001) and a non-significant increase in adults aged 50 years and over (from 12.5% to 17.8%, *p*-value: 0.004) when using the Holm–Bonferroni significance-level correction.

The analysis of the cumulative percentage obtained by summing the serotypes corresponding to the different vaccine formulations in each age group showed a trend towards increased protection against IPD, caused by the additional serotypes included in these higher-valence vaccines, independently of the age group or period. This occurred mainly due to the addition of serotypes 19A (*n* = 637/2763, 23.0%) and 3 (*n* = 333/2763, 12.0%), the most frequently identified in this study. Although there is a percentage increase in the contribution of serotypes contained in vaccines with a higher valence than PCV10 between the pre-COVID and COVID periods, both for children and adults over 50 years of age, considering the level of significance corrected for the multiplicity of hypothesis tests (≤0.002), none of these increases would be considered statistically significant (Table 3).

### 3.3. Antimicrobial Resistance

**Figure 2:**
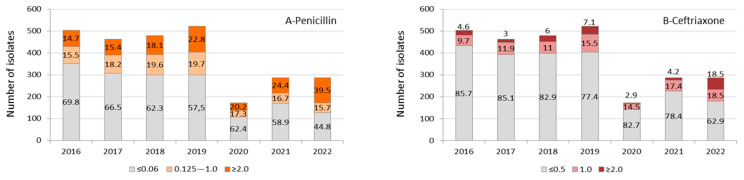
Invasive *S. pneumoniae* (*n* = 2.714) isolates’ distribution and proportion of values for minimum inhibitory concentration (μg/mL) to penicillin (**A**) and ceftriaxone (**B**) by year. Data from the year 2022 corresponded to a period from January to May. The antimicrobial susceptibility profile was not determined in 57 *S. pneumoniae* isolates identified by molecular methodology.

Except for the year 2020 due to the low number of isolates, we observed a gradual increase in the proportion of *S. pneumoniae* isolates with MIC values for penicillin ≥0.125 μg/mL and ceftriaxone ≥1.0 μg/mL during the period studied, reaching a proportion of 55.2% and 37% of isolates, respectively, by the first months of 2022. We also observed an increasing trend towards higher levels of MIC values (≥2.0 μg/mL) for both antimicrobials, especially when analyzing the COVID-19 year of 2021 (24.4% for penicillin) and 2022 (39.5% for penicillin and 18.5% for ceftriaxone) (Figure 2A,B).

The non-susceptibility rates to penicillin and ceftriaxone were higher for patients with meningitis than for patients with other clinical forms in both age groups and periods. They were also higher in the COVID period than in the pre-COVID period. The statistical comparison showed that the levels were significantly higher in the COVID period, particularly for cases of clinical forms other than meningitis in both age groups, except for ceftriaxone in adults (Table 4).

Except for levofloxacin, the overall trend of increased non-susceptibility rates in the COVID-19 period compared to the pre-COVID-19 period was also documented for the non-beta-lactam antimicrobials erythromycin, clindamycin, tetracycline, and sulfamethoxazole-trimethoprim, increasing multidrug resistance (Table 5). All *S. pneumoniae* isolates were susceptible to vancomycin.

**Figure 3:**
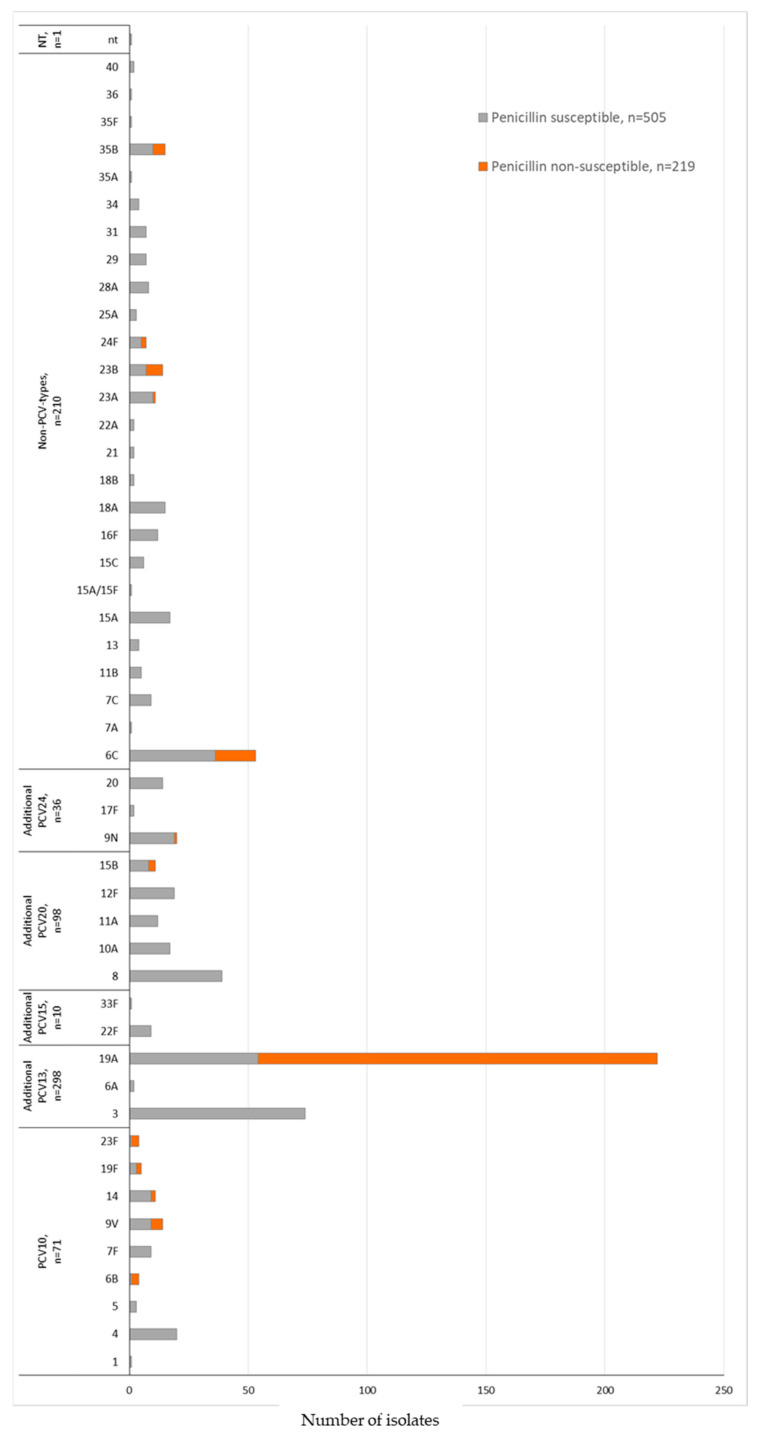
Penicillin susceptibility profile and serotyping distribution of invasive *S. pneumoniae* (N = 724) isolates in the COVID-19 period (2020 February–2022 May) by PCV formulations (PCV10; additional serotypes PCV13, PCV15, PCV20, or PCV24; non-PCVs types; and non-typable). Penicillin non-susceptibility corresponding to *S. pneumoniae* isolates with MIC ≥ 0.125 μg/mL according to the CLSI, 2022. The susceptibility profile was not determined in 43 *S. pneumoniae* isolates identified by molecular methodology.

The serotype distribution according to penicillin non-susceptibility (MIC ≥ 0.125 μg/mL) in the COVID-19 period identified 13 serotypes. We also observed that 89.9% (*n* = 197/219) of the *S. pneumoniae* isolates belonged to the non-PCV10 types 19A (*n* = 168), 6C (*n* = 17), 23B (*n* = 7), and 35B (*n* = 5) (Figure 3 and Table 6). The MDR was associated mainly with non-PCV10 types 19A (*n* = 194, 87.4%) and 6C (*n* = 43, 81.1%) (Table 6). Serotype 3, the second most prevalent serotype, presented 100% susceptibility to penicillin and ceftriaxone and displayed low non-susceptibility rates to the other antimicrobials or multidrug resistance (Table 6).

## 4. Discussion

Limited information on *Streptococcus pneumoniae* laboratory data is available from emerging countries recovered from the COVID-19 pandemic era. This study presents a detailed analysis of a large collection of IPD isolates from Brazil, comparing pre- and COVID-19 periods, focusing on serotype antigens and antimicrobial resistance profiles. Our data indicate no emergence or epidemiological capsular switch of the pneumococcal serotype occurred, but changes in antimicrobial patterns were observed.

Studies comprising data from 30 countries, including Brazil, showed the sustained reduction in the isolation of *S. pneumoniae* [21,22,23,24] and other respiratory transmission pathogens (*H. influenzae* and *N. meningitidis*) [21,22] from severe invasive diseases during the COVID-19 pandemic, associated mainly to the introduction of containment policies, such as non-pharmacological measures and restriction of people circulation, leading to a significant reduction in life-threatening invasive diseases in many countries worldwide [21,22,23,24], a phenomenon which was also documented in our study. We observed an overall reduction of 44.6% in the invasive *S. pneumoniae* isolates received by the IAL in the COVID-19 period when compared with the pre-COVID-19 period, despite the gradual increase in invasive *S. pneumoniae* isolates during the last few months of the COVID-19 period in 2022, which coincided with the gradual loosening of the non-pharmacological measures to control virus transmission. Our study also revealed no changes in the diversity and frequency of *S. pneumoniae* serotypes between the periods and age groups analyzed, as reported in some European and Latin American countries [21,22]. 

Brazilian studies conducted on PCV10’s impact showed a great effect of this vaccine in the reduction in IPD and nasopharyngeal colonization by PCV10 types, in addition to the increase in non-PCV10 types [14,25,26,27,28]. A high diversity of non-PCV10 types was found in IPD, mainly by serotypes 3, 19A, 6C, 8, and 23A, the most prevalent in Brazil in the 2017–2019 period [14]. Our data support the consistency of a long-term PCV10 impact on IPD due to the lower prevalence of PCV10 types in the COVID-19 period in both of the age groups studied and highlights the sustained transmission of non-PCV10 types 19A, 3, and 6C during the periods studied. This was observed in both age groups but mainly in the <5-year-old group represented by 63.4% of the isolates described by our study in the COVID-19 period. 

Despite a decrease in the overall numbers, we observed a significant increase in serotype 19A during the COVID-19 pandemic period in the group of people <5 years old. Our data highlight the persistence of serotype 19A in the population, as observed in the first year of the COVID-19 pandemic in the Netherlands [29], demonstrating the ability of serotype 19A to adapt and persist within a community. The expansion of serotype 19A’s lineage clonal complex 320 associated with antimicrobial resistance has been well documented in Brazil since PCV10’s introduction in 2010 [30,31,32], suggesting that vaccination pressure added to the pressure resulting from the use of antimicrobials, such as beta-lactams, mainly in <5-year-old children, resulting in the selection of this multidrug-resistant serotype 19A lineage of pneumococcus which presents a competitive advantage for transmission and pathogenicity.

The data presented suggest that the use of conjugate vaccines with a higher valence than PCV10, licensed (PCV13, PCV15, or PCV20) or under development (PCV24), would increase protection against IPD in both of the studied populations, given that their formulation includes serotypes 19A and 3, the most prevalent serotypes in Brazil. Studies have demonstrated the presence of cross-protection between serotype 6A, an antigen present in vaccine formulations other than PCV10, and serotype 6C [33,34,35,36]. Serotype 6C was the third most prevalent serotype in Brazil described in this study, so a potential expansion in IPD protection could be obtained with the introduction of vaccines which include serotype 6A.

An important concern has arisen due to the containment measures created to block the dissemination of COVID-19. It has been argued that, by circulating pneumococcal serotypes, especially non-vaccine serotypes, there may be a possible debt in natural or innate immunity due to low colonization [10]. Also, the possibility of compromising the indirect effect of vaccination in the unvaccinated population has been argued. These factors could increase the risk for IPD by non-PCV types and PCV types after the COVID-19 pandemic era [10] and reinforce the importance of continuous surveillance of pneumococcal infections in understanding the possible future role of new vaccine formulations.

Cohen and collaborators (2021) highlighted an emergency scenario when they mentioned that the COVID-19 pandemic has led to the risk of a resurgence of vaccine-preventable diseases. This is due to the fact that low vaccination coverage, the absence of catch-up campaigns, and less exposure of individuals to viral or bacterial infections have been observed globally [10]. Data from the Brazilian National Immunization Program indicated a reduction in PCV10 coverage in Brazil since the pre-COVID period, with an aggravation in the COVID-19 period. The coverage of 81.5% between the years 2016 and 2019 (pre-COVID-19) fell to 69.9% between the years 2020 and 2022 (COVID-19) (Supplementary Figure S3). This alerts us to a risk of resurgence of PCV10 serotypes in this vaccine’s target population, with potential impairment of the indirect effect of the vaccine in the unvaccinated population.

An increase in antibiotic consumption in emerging countries, including Brazil, presenting high rates, from 2000 to 2010 has previously been reported [37]. Faced with this situation, since 2010, Brazil has introduced restrictions for over-the-counter sales of antimicrobials, determining that sales should be associated with the presentation and retention of a medical prescription, promoting control over the use of these products in the country [38]. This measure, associated with the introduction of PCVs, which contained the prevalent serotypes associated with the antimicrobial resistance causing the IPD in question, resulted in an initial reduction in the non-susceptibility rates to antimicrobials, mainly to beta-lactams, used for the treatment of pneumococcal infections [38,39,40,41].

In Brazil, studies evaluating the long-term impact of the use of PCV10 on antimicrobial resistance have demonstrated that the benefit of controlling antimicrobial resistance has impacted by pneumococcal infections caused by non-PCV10 serotypes associated with antimicrobial resistance, mainly serotypes 19A and 6C [25,30,31,39,40,42]. 

Our study showed a concerning situation with a higher rate of non-susceptibility related to beta-lactams antimicrobials, mainly in the <5-year-old group of non-meningitis infections, and other antimicrobials classes. This resulted in an elevation of multidrug-resistant *S. pneumoniae* isolates that may be related not only to the higher prevalence of non-PCV10 types 19A and 6C but also to the gross misuse of antimicrobials to avoid possible co-infections in severe cases of SARS-CoV-2 infections during the COVID-19 pandemic [13,43]. In our study. it was documented that beta-lactam non-susceptibility was associated with some risk factors such as recent antibiotic use, age (mainly in children <5 years of age), hospitalization, living in urban areas, attending daycare, and individuals with immunosuppression [44]. Antimicrobial misuse, mainly for viral infections, and the excessive use of broad-spectrum antibiotics, such ceftriaxone, were also related to the emergence of multidrug-resistant microorganisms [45].

The diversity of the emerging non-PCV10 serotypes with antimicrobial resistance may be related to many factors. The presence of pneumococcus in the nasopharynx favors its natural ability to acquire or exchange genetic material, via transformation or recombination, with other pneumococci or microorganisms, facilitating the development of antimicrobial resistance [1]. In an attempt to understand the nasopharyngeal pneumococcal carriage pattern, Weinberger and collaborators (2009) proposed a model based on the importance of the role of the biochemical structure of the capsular polysaccharide. The model proposed suggested that some serotypes, such as 19A and 6A, that presented polysaccharides less metabolically costly (with fewer carbons per repeat unit, for example) were more encapsulated, able to avoid the host immune system, and capable of persisting in the nasopharynx [46]. So, this persistence carriage pattern may explain the selection of prevalent MDR lineages of serotypes 19A and 6C in our study. Point mutations, capsular switch, and vaccine pressure are also factors related to the emergence of non-vaccine serotypes lineages associated with antimicrobial resistance [44], as well-described for the lineage 19A MDR clonal complex 320 in the post-PCV10 period in Brazil [30,31,32]. The plasticity of the pneumococcus results in a genetic structure that can determine variations in the capacity to adapt, transmit, and be virulent, which could explain the success of some non-vaccine serotypes, and future genomic studies will provide enhanced understanding of the behavior of the pneumococcal population.

This study has important limitations that should be considered. The quality of epidemiological surveillance differs between the various geographic regions of the country. Our data are obtained from laboratory-based surveillance, and they are subject to variations in the healthcare system, which may have involved restrictions on healthcare, medical prescription of antimicrobials, and also failures in sample collection, processing, reporting, and recording; therefore, our data certainly underestimate the real burden of IPD cases in Brazil.

## 5. Conclusions

This study contributes to understanding the pneumococcal antigen vaccines in circulation as well as the antibiotic resistance profile during the global spread of COVID-19 in our region. We observed no differences in serotype distribution between the studied periods; the COVID-19 period was marked by the high prevalence of serotypes 19A, 3, and 6C in both age groups and a reduction in the *S. pneumoniae* isolates received by the IAL. We also showed elevated resistance rates, mainly to penicillin and ceftriaxone for non-meningitis cases in children under 5 years of age, and these non-antimicrobial susceptibilities were related to serotypes 19A and 6C. The present data strengthen the notion that continued laboratory surveillance supports local epidemiological data in making prompt decisions for the treatment, prevention, and control of IPDs.

## Figures and Tables

**Table 1:** Distribution of and percentage change in invasive *S. pneumoniae* serotypes in <5-year-old group by period studied.

Serotypes	Period Studied			
	Pre-COVID-19 (N = 656)		COVID-19 (N = 295)	
	*n*	%	*n*	%
PCV10 types	31	4.7	11	3.7
1	1	0.2	0	0.0
4	2	0.3	0	0.0
6B	1	0.2	0	0.0
7F	6	0.9	2	0.7
9V	6	0.9	5	1.7
14	8	1.2	4	1.4
18C	1	0.2	0	0.0
19F	2	0.3	0	0.0
23F	4	0.6	0	0.0
Non-PCV10 types	625	95.3	284	96.3
**3**	**60**	**9.1**	**26**	**8.8**
6A	12	1.8	0	0.0
**6C**	**56**	**8.5**	**19**	**6.5**
7C	4	0.6	3	1.0
9N	8	1.2	3	1.0
8	13	2.0	8	2.7
10A	20	3.1	5	1.7
11A	8	1.2	3	1.0
11B	2	0.3	1	0.3
12F	19	2.9	4	1.4
13	8	1.2	0	0.0
15A	18	2.7	9	3.1
15B	14	2.1	6	2.0
15C	14	2.1	4	1.4
16F	14	2.1	4	1.4
18A	2	0.3	6	2.0
**19A**	**244**	**37.2**	**142**	**48.1**
20	1	0.2	2	0.7
21	2	0.3	1	0.3
22F	13	2.0	2	0.7
23A	17	2.6	3	1.0
23B	18	2.7	6	2.0
24F	18	2.7	5	1.7
25A	11	1.7	1	0.3
25F/25A/38	2	0.3	0	0.0
28A	4	0.6	2	0.7
29	3	0.5	5	1.7
33F	1	0.2	1	0.3
34	3	0.5	0	0.0
35B	9	1.4	5	1.7
NT *	2	0.3	1	0.3
ND ^&^	0	0.0	3	1.0
Other non-PCV10 types ^#^	5	0.8	4	1.4

The main results of the study are in bold; * Non-typeable *S. pneumoniae* isolate (obtained by Quellung reaction and PCR detection); ^&^ Not determined serotype of *S. pneumoniae* isolate (obtained by PCR detection only in non-viable isolates); ^#^ *S. pneumoniae* isolates serotypes 7A, 15F, 17F, 18B, 19B, 24B, 31, 35F, and 36, each represented by only *n* = 1 isolate in the pre-COVID-19 (2016–2020 January) or COVID-19 period (2020 February–2022 May). Serotyping was not performed in *n* = 1 not recovered *S. pneumoniae* isolate.

**Table 2:** Distribution of and percentage change in invasive *S. pneumoniae* serotypes in ≥50-year-old group by period studied.

Serotypes	Period Studied			
	Pre-COVID-19 (N = 1340)		COVID-19 (N = 472)	
	*n*	%	*n*	%
PCV10 types	175	13.1	66	14
1	3	0.2	1	0.2
4	63	4.7	22	4.7
5	13	1.0	3	0.6
6B	9	0.7	5	1.1
7F	33	2.5	8	1.7
9V	17	1.3	10	2.1
14	15	1.1	8	1.7
18C	5	0.4	0	0.0
19F	10	0.8	5	1.1
23F	7	0.5	4	0.9
Non-PCV10 types	1165	86.9	406	86.0
**3**	**190**	**14.2**	**57**	**12.1**
6A	13	1.0	2	0.4
**6C**	**106**	**7.9**	**37**	**7.8**
7A	2	0.1	0	0.0
7C	6	0.4	6	1.3
8	76	5.7	34	7.2
9N	33	2.5	18	3.8
10A	30	2.2	14	3.0
11A	45	3.4	10	2.1
11B	4	0.3	4	0.8
12F	50	3.7	19	4.0
13	12	0.9	4	0.9
15A	32	2.4	8	1.7
15B	10	0.8	5	1.1
15C	11	0.8	2	0.4
16F	42	3.1	8	1.7
17F	12	0.9	1	0.2
18A	17	1.3	9	1.9
18B	2	0.1	2	0.4
**19A**	**167**	**12.5**	**84**	**17.8**
20	33	2.5	13	2.8
21	3	0.2	1	0.2
22A	1	0.1	2	0.4
22F	43	3.2	7	1.5
23A	58	4.3	9	1.9
23B	31	2.3	8	1.7
24F	17	1.3	2	0.4
25A	14	1.0	2	0.4
28A	11	0.8	6	1.3
29	10	0.8	2	0.4
31	2	0.1	6	1.3
34	13	1.0	4	0.8
35A	2	0.1	1	0.2
35B	37	2.8	10	2.1
35C	2	0.1	0	0.0
35F	14	1.0	0	0.0
36	1	0.1	1	0.2
40	2	0.1	2	0.4
NT *	2	0.1	0	0.0
ND ^&^	0	0.0	5	1.1
Other non-PCV10 types ^#^	9	0.7	1	0.2

The main results of the study are in bold; * Non-typeable *S. pneumoniae* isolate (obtained by Quellung reaction and PCR detection); ^&^ Not determined serotype of *S. pneumoniae* isolate (obtained by PCR detection only in non-viable isolates); ^#^ *S. pneumoniae* isolates serotypes 6D, 7C/7B/40, 15A/15F, 15F, 19C, 25F/25A/38, 28F, 33F, 38, and 47F, each represented by only *n* = 1 in the pre-COVID-19 (2016–2020 January) or COVID-19 period (2020 February–2022 May). Serotyping was not performed in *n* = 7 not recovered *S. pneumoniae* isolates.

**Table 3:** Distribution of the invasive *S. pneumoniae* serotypes by vaccine formulations (PCV10, PCV13, PCV15, PCV20, and PCV24) by age group. Hypothesis testing of the difference in distribution between periods by formulation and age group.

Serotypes by Vaccine Formulations	<5 Years Old					≥50 Years Old				
	Pre-COVID-19		COVID-19			Pre-COVID-19		COVID-19		
	N = 656 ^#^	Cum.%	N = 295	Cum.%	*p*-Value ^$^	N = 1.340 ^#^	Cum.%	N = 472	Cum.%	*p*-Value ^$^
PCV10 *	31	4.7	11	3.7	0.489	175	13.1	66	14.0	0.611
PCV13	347	52.9	179	60.7	0.026	545	40.7	209	44.3	0.171
PCV15	361	55.0	182	61.7	0.055	589	44.0	216	45.8	0.487
PCV20	435	66.3	208	70.5	0.201	800	59.7	298	63.1	0.189
PCV24 **	444	67.7	214	72.5	0.133	878	65.5	330	69.9	0.082
NVT ^&^	212	32.3	81	27.5	0.133	462	34.5	142	30.1	0.082

^#^ Serotyping was not performed in eight not recovered isolates (one from the pre-COVID-19 period in the <5-year-old group and seven from the pre-COVID-19 period in the ≥50-year-old group); ^$^ statistical significance *p* ≤ 0.002; * PCV10 is the vaccine formulation that has been available for childhood vaccination in Brazil since 2010; ** vaccine formulation under development; and ^&^ non-vaccine serotypes according to the described vaccine formulations, including serotype 6C.

**Table 4:** Non-susceptibility rates to penicillin and ceftriaxone of invasive *S. pneumoniae* isolates from meningitis and non-meningitis clinical diagnoses by age group and study period.

Non- Susceptibility *	<5 Years Old							≥50 Years Old						
	Pre-COVID-19 ^&^			COVID-19 ^&^				Pre-COVID-19^&^			COVID-19 ^&^			
	N ^#^	*n*	%	N ^#^	*n*	%	*p*-Value ^$^	N ^#^	*n*	%	N ^#^	*n*	%	*p*-Value ^$^
Meningitis														
Penicillin	263	128	48.7	70	45	64.3	0.020	437	139	31.8	99	41	41.4	0.068
Ceftriaxone		67	25.5		23	32.9	0.216		52	11.9		17	17.2	0.157
Non-meningitis														
Penicillin	391	80	20.5	214	84	39.3	**<0.001**	899	56	6.2	341	49	14.4	**<0.001**
Ceftriaxone		36	9.2		41	19.2	**<0.001**		33	3.7		16	4.7	0.410
Total														
Penicillin	654	208	31.8	284	129	45.4	**<0.001**	1336	195	14.6	440	90	20.5	0.004
Ceftriaxone		103	15.7		64	22.5	0.013		85	6.4		33	7.5	0.404

* Non-susceptible: intermediate plus resistant criteria according to the CLSI, 2022; ^&^ study period: pre-COVID-19 (2016–2020 January) and COVID-19 (2020 February–2022 May); ^#^ susceptibility profile was not performed in 57 not recovered isolates (*n* = 15 from meningitis [*n* = 3 in the <5-year-old group in the COVID-19 period, *n* = 2 in the ≥50-year-old group in the pre-COVID-19 period, and *n* = 10 in the ≥50-year-old group in the COVID-19 period] and *n* = 42 from non-meningitis [*n* = 3 in the <5-year-old group in the pre-COVID-19 period, *n* = 8 in the <5-year-old group in the COVID-19 period, *n* = 9 in the ≥50-year-old group in the pre-COVID-19 period, and *n* = 22 in the ≥50-year-old group in the COVID-19 period]); and ^$^ statistical significance *p* ≤ 0.002 (in bold).

**Table 5:** Non-susceptibility * rates to non-beta-lactam antimicrobials of invasive *S. pneumoniae* isolates per study period ^$^.

Antimicrobial	N. Tested	Non-Susceptible Isolates		*p*-Value ^#^
		*n*	%	
Erythromycin				
Pre-COVID-19	1989	672	33.8	**<0.001**
COVID-19	724	343	47.4	
Clindamycin				
Pre-COVID-19	1778	495	27.8	**<0.001**
COVID-19	724	271	37.4	
Tetracycline				
Pre-COVID-19	866	380	43.9	**<0.001**
COVID-19	724	434	59.9	
Trimethoprim-Sulfamethoxazole				
Pre-COVID-19	1967	760	38.6	**<0.001**
COVID-19	724	351	48.5	
Levofloxacin				
Pre-COVID-19	868	4	0.5	0.131 **
COVID-19	724	0	0	
MDR ^&^				
Pre-COVID-19	1990	498	25	**<0.001**
COVID-19	724	315	43.5	

* Non-susceptible: intermediate plus resistant criteria according to the CLSI, 2022; ^$^ study period: pre-COVID-19 (2016–2020 January) and COVID-19 (2020 February–2022 May); ^#^ statistical significance *p* ≤ 0.002 (in bold); ** Fisher’s exact test; and **^&^** multidrug-resistant: non-susceptibility to at least three antimicrobial classes including penicillin. All the isolates are susceptible to vancomycin.

**Table 6:** Rates of antimicrobial non-susceptibility * of invasive *S. pneumoniae* (*n* = 720) isolates by pneumococcal vaccine formulation in the COVID-19 period.

PCV Formulation	Serotype	Total	MDR ^$^		PEN Non-Susceptible		CRO Non-Susceptible		ERY Non-Susceptible		CLI Non-Susceptible		SXT Non-Susceptible		TET Non-Susceptible	
			*n*	%	N	%	*n*	%	*n*	%	*n*	%	*n*	%	*n*	%
PCV10	9V	14	13	92.9	5	35.7	2	14.3	13	92.9	0	0.00	13	92.9	13	92.9
	23F	4	3	75.0	3	75.0	2	50.0	3	75.0	1	25.0	2	50.0	4	100
	6B	4	2	50.0	3	75.0	0	0.0	2	50.0	2	50.0	3	75.0	3	75.0
	19F	5	2	40.0	2	40.0	0	0.0	2	40.0	1	20.0	1	20.0	3	60.0
	14	11	2	18.2	2	18.2	1	9.1	9	81.8	3	27.3	4	36.4	6	54.5
	4	20	0	0.0	0	0.0	0	0.0	0	0.0	0	0.0	1	5.0	6	30.0
	5	3	0	0.0	0	0.0	0	0.0	0	0.0	0	0.0	3	100	2	66.7
	7F	9	0	0.0	0	0.0	0	0.0	0	0.0	0	0.0	0	0.0	1	11.1
Additional PCV13	**19A**	**222**	**194**	**87.4**	**168**	**75.7**	**91**	**41.0**	**199**	**89.6**	**175**	**78.8**	**211**	**95**	**188**	**84.7**
	3	74	2	2.7	0	0.0	0	0.0	3	4.1	3	4.1	3	4.1	13	17.6
	6A	2	0	0.0	0	0.0	0	0.0	0	0.0	0	0.0	1	50.0	2	100
Additional PCV15	33F	1	1	100	0	0.0	0	0.0	1	100	1	100	0	0.0	1	100
	22F	9	0	0.0	0	0.0	0	0.0	0	0.0	0	0.0	0	0.0	4	44.4
Additional PCV20	15B	11	7	63.6	3	27.3	0	0.0	7	63.6	1	9.1	10	90.9	9	81.8
	10A	17	2	11.8	0	0.0	0	0.0	2	11.8	2	11.8	5	29.4	11	64.7
	8	39	2	5.1	0	0.0	0	0.0	2	5.1	2	5.1	1	2.6	11	28.2
	12F	19	1	5.3	0	0.0	0	0.0	1	5.3	1	5.3	8	42.1	2	10.5
	11A	12	0	0.0	0	0.0	0	0.0	0	0.0	0	0.0	0	0.0	1	8.3
Additional PCV24	20	14	11	78.6	0	0.0	0	0.0	11	78.6	11	78.6	6	42.9	14	100
	9N	20	5	25.0	1	5.0	0	0.0	6	30.0	4	20.0	10	50.0	13	65.0
	17F	2	0	0.0	0	0.0	0	0.0	0	0.0	0	0.0	0	0.0	2	100
Non- PCV types	**6C**	**53**	**43**	**81.1**	**17**	**32.1**	**0**	**0.0**	**46**	**86.8**	**41**	**77.4**	**7**	**13.2**	**43**	**81.1**
	24F	7	5	71.4	2	28.6	0	0.0	7	100	7	100	2	28.6	5	71.4
	23A	11	8	72.7	1	9.1	0	0.0	8	72.7	8	72.7	1	9.1	9	81.8
	40	2	1	50.0	0	0.0	0	0.0	2	100	1	50.0	0	0.0	2	100
	15C	6	2	33.3	0	0.0	0	0.0	4	66.7	2	33.3	4	66.7	4	66.7
	15A	17	4	23.5	0	0.0	0	0.0	4	23.5	3	17.6	11	64.7	11	64.7
	23B	14	3	21.4	7	50	0	0.0	1	7.1	0	0.0	14	100	5	35.7
	16F	12	2	16.7	0	0.0	0	0.0	2	16.7	2	16.7	9	75.0	5	41.7
	7C	9	0	0.0	0	0.0	0	0.0	0	0.0	0	0.0	6	66.7	6	66.7
	11B	5	0	0.0	0	0.0	0	0.0	0	0.0	0	0.0	0	0.0	4	80.0
	13	4	0	0.0	0	0.0	0	0.0	0	0.0	0	0.0	3	75.0	3	75.0
	15A/15F	1	0	0.0	0	0.0	0	0.0	0	0.0	0	0.0	1	100	1	100
	18A	15	0	0.0	0	0.0	0	0.0	0	0.0	0	0.0	2	13.3	5	33.3
	18B	2	0	0.0	0	0.0	0	0.0	0	0.0	0	0.0	0	0.0	2	100
	21	2	0	0.0	0	0.0	0	0.0	0	0.0	0	0.0	0	0.0	1	50.0
	22A	2	0	0.0	0	0.0	0	0.0	0	0.0	0	0.0	0	0.0	1	50.0
	25A	3	0	0.0	0	0.0	0	0.0	0	0.0	0	0.0	1	33.3	1	33.3
	28A	8	0	0.0	0	0.0	0	0.0	0	0.0	0	0.0	2	25.0	6	75.0
	29	7	0	0.0	0	0.0	0	0.0	0	0.0	0	0.0	0	0.0	4	57.1
	31	7	0	0.0	0	0.0	0	0.0	0	0.0	0	0.0	0	0.0	2	28.6
	34	4	0	0.0	0	0.0	0	0.0	0	0.0	0	0.0	2	50.0	0	0.0
	35A	1	0	0.0	0	0.0	0	0.0	0	0.0	0	0.0	0	0.0	1	100
	35B	15	0	0.0	5	33.3	1	6.7	8	53.3	0	0.0	4	26.7	3	20.0
	36	1	0	0.0	0	0.0	0	0.0	0	0.0	0	0.0	0	0.0	1	100

The main results of the study are in bold; * Non-susceptibility, intermediate plus resistant criteria according to the CLSI, 2022; ^$^ multidrug-resistant: non-susceptibility to at least three antimicrobial classes; PEN, penicillin; CRO, ceftriaxone; ERY, erythromycin; CLI, clindamycin; SXT, sulfamethoxazole-trimethoprim; and TET, tetracycline. In the COVID-19 period (2020 February–2022 May), serotypes 1, 7A, 35F, and NT (*n* = 1 each) were susceptible to all antimicrobials, and all *S. pneumoniae* isolates were susceptible to levofloxacin and vancomycin.

## Data Availability

The data presented in the study are available upon request to the corresponding author.

## Supplementary Materials

The following supporting information can be downloaded at https://www.mdpi.com/article/10.3390/microorganisms12020401/s1: Figure S1. Confirmed cases and incidence of pneumococcal meningitis in Brazil from 2010 to 2022 (N = 12,406). Source: Information System for Notifiable Diseases—Sinan, Ministry of Health, Brazil (http://tabnet.datasus.gov.br/cgi/deftohtm.exe?sinannet/cnv/meninbr.def, accessed on 3 August 2023); Figure S2. Flow chart describing the methodology used in this study; Figure S3. Vaccination coverage for PCV10 in Brazil from 2013 to 2022. Source: National Immunization Program Information System (http://tabnet.datasus.gov.br/cgi/webtabx.exe?bd_pni/cpnibr.def, accessed on 7 August 2023). In 2010 Brazil introduced the PCV10 into the national childhood immunization program; and Table S1. Epidemiologic data from invasive *S. pneumoniae* isolates by age group and period studied.
