# Supplementary material for: Serotype Distribution and Antimicrobial Susceptibility Pattern of Streptococcus pneumoniae in COVID-19 Pandemic Era in Brazil
Source: Microorganisms. 2024 Feb 17;12(2):401. doi: 10.3390/microorganisms12020401 (PMC10893029; doi:10.3390/microorganisms12020401)
Supplement: Supplementary file 1 [file microorganisms-12-00401-s001.zip › Table S1.pdf]

Table S1. Epidemiologic data from the invasive *S. pneumoniae* isolates by age group and period studied.

Age group	Epidemiologic data	Pre-COVID-19 (January 2016-January 2020) N=2004	COVID-19 (February 2020-May 2022) N=767	Total N=2771
<5 years	Total	657 (69.0%)	295 (31.0%)	952
	Mean age	1.2 years (1 month-4 years)	8 months (1 month-4 years)	-
	Clinical diagnosis:			
	Meningitis	263 (78.3%)	73 (21.7%)	336
	Non-meningitis	394 (64.0%)	222 (36.0%)	616
≥50 years	Total	1347 (74.1%)	472 (25.9%)	1819
	Mean age	74.5 years (50-99 years)	72.2 years (50-99 years)	-
	Clinical diagnosis:			
	Meningitis	439 (80.1%)	109 (19.9%)	548
	Non-meningitis	908 (71.4%)	363 (28.6%)	1271
