# Supplementary figures and images for: Serotype Distribution and Antimicrobial Susceptibility Pattern of Streptococcus pneumoniae in COVID-19 Pandemic Era in Brazil
Source: Microorganisms. 2024 Feb 17;12(2):401. doi: 10.3390/microorganisms12020401 (PMC10893029; doi:10.3390/microorganisms12020401)

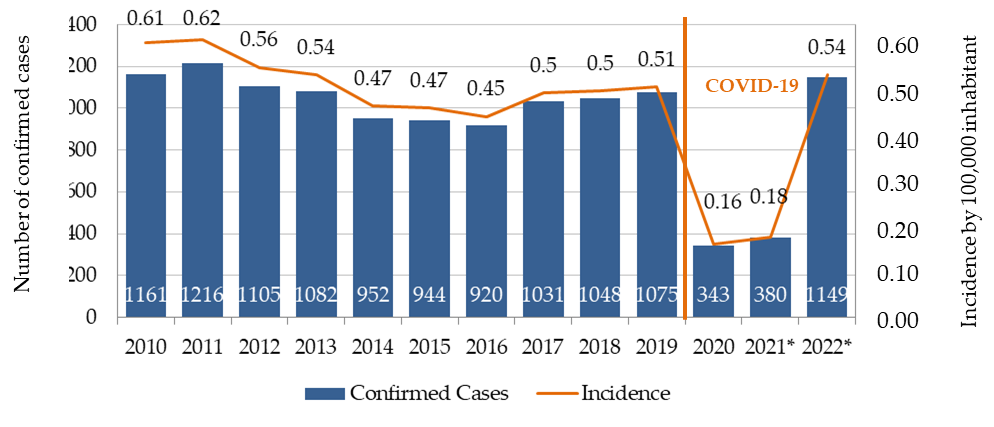

Supplement: Supplementary file 1 [file microorganisms-12-00401-s001.zip › Figure S1 2212.tif]

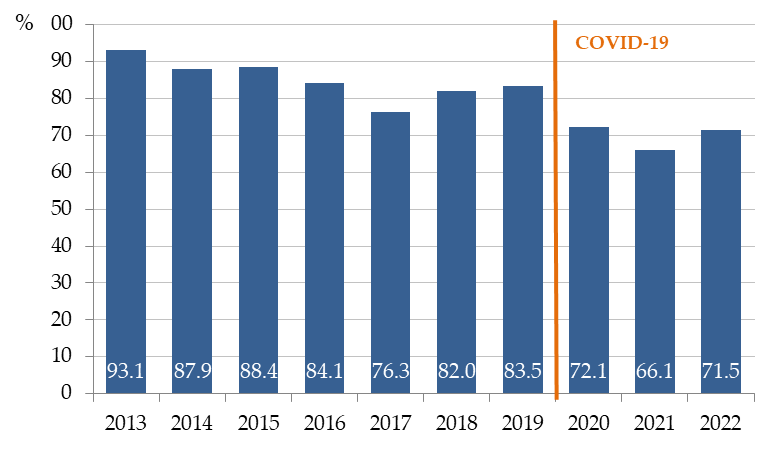

Supplement: Supplementary file 1 [file microorganisms-12-00401-s001.zip › Figure S3 2212.tif]
